# Bioinformatics data supporting revelatory diversity of cultivable thermophiles isolated and identified from two terrestrial hot springs, Unkeshwar, India

**DOI:** 10.1016/j.dib.2016.04.038

**Published:** 2016-04-23

**Authors:** Bhagwan N. Rekadwad, Chandrahasya N. Khobragade

**Affiliations:** School of Life Sciences, Swami Ramanand Teerth Marathwada University, Nanded, Maharashtra 431606, India

**Keywords:** DNA sequencing, ENDMEMO, GC content, DNA signatures, Unkeshwar hot springs

## Abstract

A total of 21 thermophilic bacteria were isolated and identified using 16S rRNA gene sequencing method. Sequences were submitted to NCBI website. Short DNA sequences JN392966–JN392972; KC120909–KC120919; KM998072–KM998074 and KP053645 strains were downloaded from NCBI BioSample database. ENDMEMO GC calculating tool was used for calculation of maximum, minimum and average GC percentage and graphical representation of GC content. Data generated indicate 20 short DNA sequences have maximum GC content ranged from 60% to 100% with an average GC content 52.5–59.8%. It is recorded that *Bacillus* sp. W7, *Escherichia coli* strain NW1 and *Geobacillus thermoleovorans* strain rekadwadsis strains showed GC content maximum up to 70%; *Actinobacterium* EF_NAK1-7 up to 85.7%, while *Bacillus megaterium* and *E. coli* strain NW2 showed GC content maximum to 100%. Digital data on thermophilic bacteria isolated from Unkeshwar hot springs would be useful for interpretation of presence of biodiversity in addition to phenotypic, physiological characteristics and data generated through 16S rRNA gene sequencing technology.

**Specifications Table**

**Table:** 

Subject area	*Microbiology*
More specific subject area	*Bioinformatics*
Type of data	*Table, graphs*
How data was acquired	*Outsourcing of NCBI site*
Data format	*Raw and Analyzed*
Experimental factors	*GC content were determined through graphical representation*
Experimental features	*Short DNA sequences (16S rRNA) were used*
Data source location	Bioinformatics Research Laboratory, School of Life Sciences, S. R. T. M. University, Nanded, India
Data accessibility	Data available within this article

## Value of the data

• Data provides information of the GC content of bacteria isolated from Unkeshwar hot springs.
• This data would be valuable for quantitative study microbial diversity of exists in Unkeshwar hot spring.
• This data would be valuable for further studies of the molecular mechanism underlying adaptation of thermophiles at high temperatures.

## 1 Data

This paper contains data on GC content of 21 thermophilic bacteria isolated and identified by us. Short DNA sequences were submitted to NCBI repository under the accession number JN392966–JN392972; KC120909–KC120919; KM998072–KM998074 and KP053645. Maximum and average GC percent of 21 thermophilic bacteria in short DNA sequences using ENDMEMO software was calculated. See also *NCBI repository* http://www.ncbi.nlm.nih.gov/nuccore [1], [2].

## 2 Experimental design, materials and methods

**Fig. 1:**
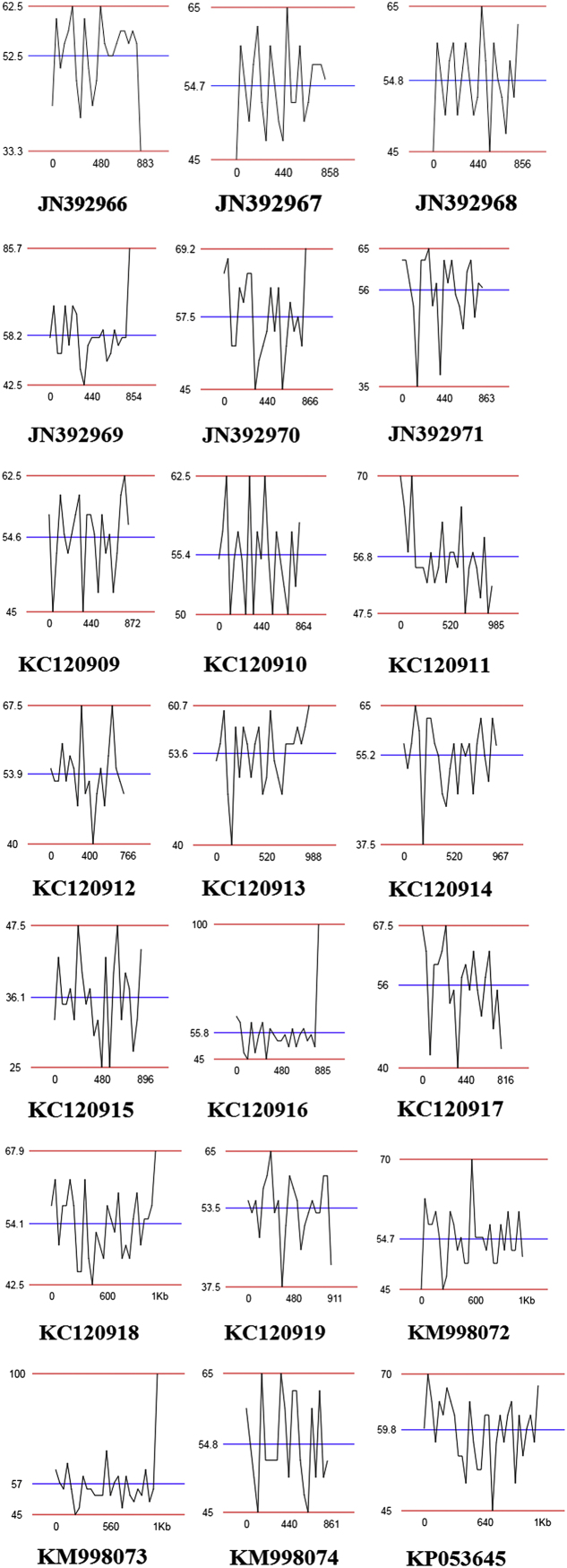
GC percentage in short DNA sequences of thermophiles (JN392966–JN392972; KC120909–KC120919; KM998072–KM998074 and KP053645) isolated from Unkeshwar hot springs.

Short DNA sequences of JN392966–JN392972; KC120909–KC120919; KM998072–KM998074 and KP053645 strains downloaded in FASTA format from NCBI BioSample database (Table 1). Using ENDMEMO GC calculating tool, GC content in percent was calculated for thermophilic bacteria. Short DNA sequence i.e. 16S rRNA gene was used for drawing GC distribution/GC graphs. ENDMEMO GC plotter showed pattern of GC distribution in DNA sequence showed through graphical representations (Fig. 1). GC pattern Graph indicates data on maximum, average and minimum GC content distribution in DNA sequence.

## Figures and Tables

**Table 1:** GC content in short DNA sequences (16S rRNAs) of thermophiles isolated and identified Unkeshwar hot springs.

**Serial no.**	**Species**	**Accession numbers**	**Maximum GC percentage**	**Total GC percentage**
1	*Naxibacter* sp. AF_NAK1-3	JN392966	62.5	52.5
2	*Bacillus licheniformis*	JN392967	65	54.7
3	*Brevibacillus borstelensis*	JN392968	65	54.8
4	*Actinobacterium* EF_NAK1-7	JN392969	85.7	58.2
5	*Brevibacillus* sp. EF_TYK1-4	JN392970	69.2	57.5
6	*Bacillus* sp. EF_TYK1-5	JN392971	65	56
7	*Bacillus pumilus*	KC120909	62.5	54.6
8	*Brevibacillus brevis*	KC120910	62.5	55.4
9	*Bacillus* sp. W7	KC120911	70	56.8
10	*Burkholderia* sp. W11	KC120912	67.5	53.9
11	*Pseudomonas pseudoalcaligenes*	KC120913	60.7	53.6
12	*Brevundimonas diminuta*	KC120914	65	55.2
13	*Acinetobacter baumannii*	KC120915	47.5	36.1
14	*Bacillus megaterium*	KC120916	100	55.8
15	*Bacillus* sp. W3	KC120917	67.5	56
16	*Alcaligenes* sp. U1(2013)	KC120918	67.9	54.1
17	*Brevibacillus* sp. NAK1-14	KC120919	65	53.5
18	*Escherichia coli* strain NW1	KM998072	70	54.7
19	*Escherichia coli* strain NW2	KM998073	100	57
20	*Escherichia coli* strain NW3	KM998074	65	54.8
21	*Geobacillus thermoleovorans* strain rekadwadsis	KP053645	70	59.8

