# Supplementary figures and images for: Bioinformatics data supporting revelatory diversity of cultivable thermophiles isolated and identified from two terrestrial hot springs, Unkeshwar, India
Source: Data Brief. 2016 Apr 23;7:1511–4. doi: 10.1016/j.dib.2016.04.038 (PMC4857395; doi:10.1016/j.dib.2016.04.038)

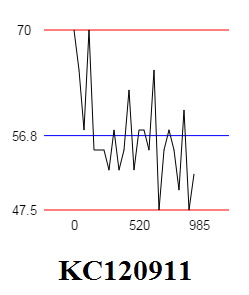

Supplement: Supplementary file 2 — Supplementary material [file mmc2.zip › KC120911.png]

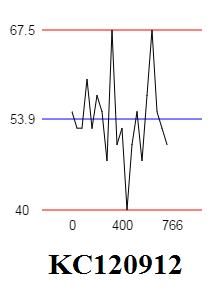

Supplement: Supplementary file 2 — Supplementary material [file mmc2.zip › KC120912.png]

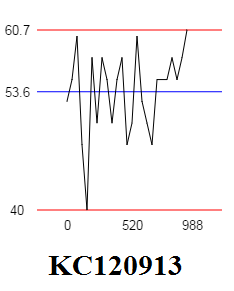

Supplement: Supplementary file 2 — Supplementary material [file mmc2.zip › KC120913.png]

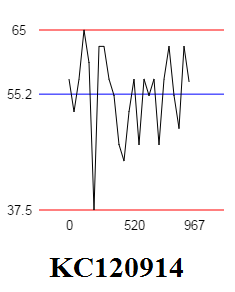

Supplement: Supplementary file 2 — Supplementary material [file mmc2.zip › KC120914.png]

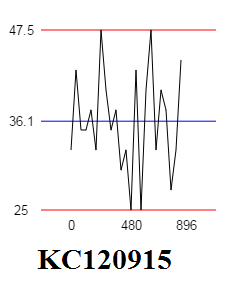

Supplement: Supplementary file 2 — Supplementary material [file mmc2.zip › KC120915.png]

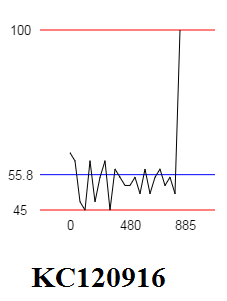

Supplement: Supplementary file 2 — Supplementary material [file mmc2.zip › KC120916.png]

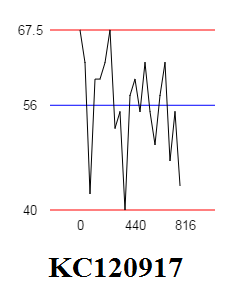

Supplement: Supplementary file 2 — Supplementary material [file mmc2.zip › KC120917.png]

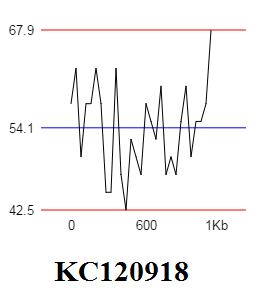

Supplement: Supplementary file 2 — Supplementary material [file mmc2.zip › KC120918.png]

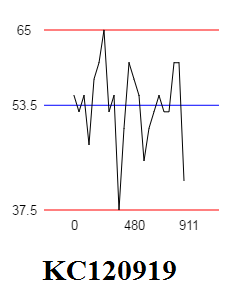

Supplement: Supplementary file 2 — Supplementary material [file mmc2.zip › KC120919.png]

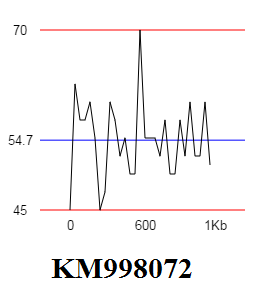

Supplement: Supplementary file 2 — Supplementary material [file mmc2.zip › KM998072.png]

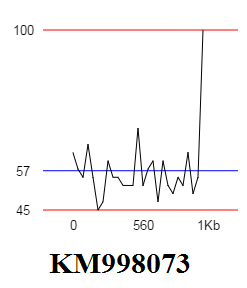

Supplement: Supplementary file 2 — Supplementary material [file mmc2.zip › KM998073.png]

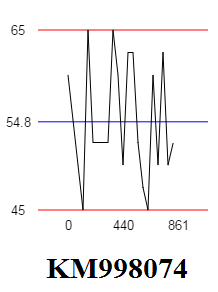

Supplement: Supplementary file 2 — Supplementary material [file mmc2.zip › KM998074.png]

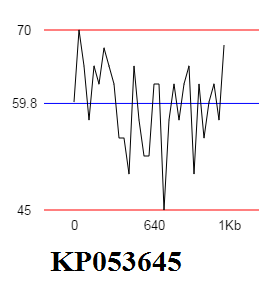

Supplement: Supplementary file 2 — Supplementary material [file mmc2.zip › KP053645.png]

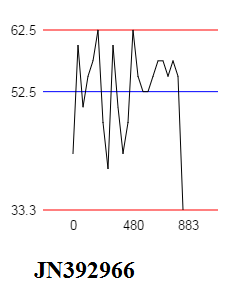

Supplement: Supplementary file 2 — Supplementary material [file mmc2.zip › JN392966.png]

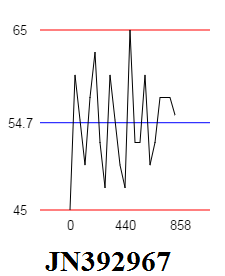

Supplement: Supplementary file 2 — Supplementary material [file mmc2.zip › JN392967.png]

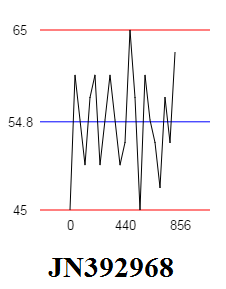

Supplement: Supplementary file 2 — Supplementary material [file mmc2.zip › JN392968.png]

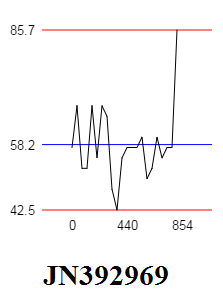

Supplement: Supplementary file 2 — Supplementary material [file mmc2.zip › JN392969.png]

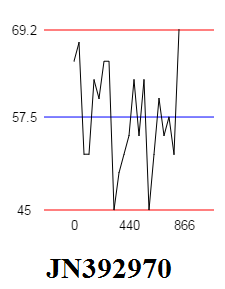

Supplement: Supplementary file 2 — Supplementary material [file mmc2.zip › JN392970.png]

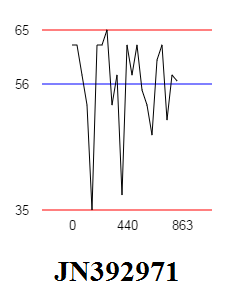

Supplement: Supplementary file 2 — Supplementary material [file mmc2.zip › JN392971.png]

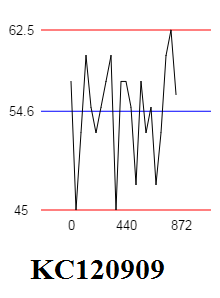

Supplement: Supplementary file 2 — Supplementary material [file mmc2.zip › KC120909.png]

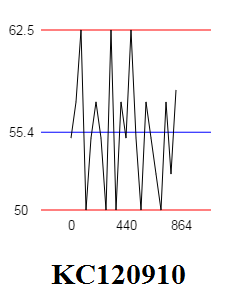

Supplement: Supplementary file 2 — Supplementary material [file mmc2.zip › KC120910.png]
